# Lonely

**Published:** 1888-08-04

**Authors:** 


### LONELY.

I'M thanking you kindly for all you've said
To a poor, rough man who is crushed to the ground,
And lonesome for want of the one that's dead.
But you've known sorrow, too, sir, I'll be bound.
Maybe, although you're a scholar, and all,
You can understand a poor chap like me.
Nell was better nor I, and a maid at the hall,
But, "I'll love ye for ever, Bob," says she.

I lived a bit wild till I saw in her face
She was grieving main for my ways; and then
I never went into a drinking-place,
Nor roughed it about with the other men.
Talk of God's angels! she was one,
And somehow the pick of them all to me.
I would kneel by her side when the day was done,
And, "I'll love ye for ever, Bob," says she.

Then she came to her time, and my lass must die—
Leave her new-born babe—and she sweetly smiled.
"We've been happy together, Bob, you and I,
Give me a kiss. Let me see the child."
So she's gone from me now! Though she could not stay
To whisper it morning and night to me,
I know she is loving me far away,
For, "I'll love ye for ever, Bob," said she.

